# Genetic resources of vegetable crops: from breeding non-traditional crops to functional food

**DOI:** 10.18699/VJ21.049

**Published:** 2021-07

**Authors:** Yu.V. Fotev, A.M. Artemyeva, O.A. Zvereva

**Affiliations:** Central Siberian Botanical Garden of the Siberian Branch of the Russian Academy of Sciences, Novosibirsk, Russia Novosibirsk State Agrarian University, Novosibirsk, Russia; Federal Research Center the N.I. Vavilov All-Russian Institute of Plant Genetic Resources (VIR), St. Petersburg, Russia; Federal Research Center the N.I. Vavilov All-Russian Institute of Plant Genetic Resources (VIR), St. Petersburg, Russia

**Keywords:** introduction, breeding, underutilized vegetable crops, functional food, functional food ingredients, интродукция, селекция, нетрадиционные овощные культуры, функциональные продукты питания, функциональные пищевые ингредиенты

## Abstract

In this review, the authors considered the promising species of vegetable crops for introduction and breeding in the Russian Federation. An attempt was made to assess the possibilities of their breeding improvement from the standpoint of the presence of traits that limit large-scale production. Species that could potentially serve as sources of a high content of functional food ingredients (FFI) have been identified and characterized. For the successful introgression of these species in the Russian Federation, we proposed the methodological approaches including the assessment of the potential cold resistance of thermophilic crops in the mature male gametophyte in vitro (e.g., asparagus bean). The increase in the biodiversity of vegetable plants and improving of their nutritional value should be recognized as one of the main tasks, along with the growth of crop productivity. It is proposed to use the ratio of the total number of the registered cultivars of a particular crop to the number of years since the first cultivar of that crop has been included in the State Register of Breeding Achievements Admitted for Use as a measure of demand. It is advisable to formalize the trait “high content of FFI” in crops, taking as a basis, for example, a 2–4-fold excess of the content of any FFI or their complex in a cultivar over the crop’s standard (reference) value. Such varieties should be included in the State Register of Breeding Achievements Approved for Use as a separate list. The purpose of their separation in the State Register is to ensure the potential interest of investors and business structures in the sale of functional food on the market. The paper discusses in detail the most promising species of introduced vegetable crops from five families (Brassicaceae, Amaranthaceae, Solanaceae, Leguminosae, Cucurbitaceae). The following species are proposed as potential sources of high FPI content: Brassica oleracea ssp. oleracea, B. oleraceae var. alboglabra, B. rapa ssp. chinensis, B. rapa ssp. narinosa, B. rapa ssp. nipposinica, B. rapa ssp. rapa, B. juncea, Cochlearia officinalis, Lepidium sativum, Amaranthus caudatus, A. cruentus, A. hypochondriacus, A. dubius, A. tricolor, A. lividus, species in the genus Physalis L., Momordica charantia, Benincasa hispida, Cucumis metuliferus, Vigna unguiculata

## Abstract (ru)

Рассмотрены перспективные для интродукции в России виды овощных культур и cделана попытка оценить возможности их селекционного улучшения по признакам, ограничивающим масштабное производство этих культур. Обсуждаются методические подходы, которые включают в себя признаки, способствующие успешной интрогрессии данных видов в РФ, и направлены на преодоление барьеров на пути расширения сортимента выращиваемых культур. На примере вигны предлагается проводить оценку потенциальной холодостойкости теплолюбивых культур в фазе зрелого мужского гаметофита in vitro. Одной из главных задач, наравне с ростом продуктивности культур, следует признать расширение сортимента рекомендуемых для широкого выращивания овощных растений. В качестве характеристики «степени востребованности культуры» предложено использовать показатель отношения общего числа зарегистрированных сортов по культуре к количеству лет с года включения в «Государственный реестр селекционных достижений, допущенных к использованию» первого сорта. Намечены возможные виды-источники высокого содержания функциональных пищевых ингредиентов (ФПИ) и охарактеризованы потенциальные доноры ФПИ. Целесообразно формализовать признак «высокое содержание ФПИ», приняв за основу двух-четырехкратное превышение содержания конкретных ФПИ или их комплекса в новом сорте по сравнению со стандартными сортообразцами или видами по селектируемой (интродуцируемой) культуре. Эти сорта целесообразно включать в «Госреестр…» отдельным списком. Цель такого выделения – обеспечить потенциальную заинтересованность производственников, инвесторов и бизнес-структур в реализации новых товарных позиций на рынке функциональных продуктов питания. Рассмотрены наиболее перспективные виды-интродуценты овощных культур из пяти семейств (Brassicaceae, Amaranthaceae, Solanaceae, Leguminosae, Cucurbitaceae). В качестве потенциальных источников высокого содержания ФПИ рекомендованы следующие виды: Brassica oleracea ssp. oleracea, B. oleraceae var. alboglabra, B. rapa ssp. chinensis, B. rapa ssp. narinosa, B. rapa ssp. nipposinica, B. rapa ssp. rapa, B. juncea, Cochlearia officinalis, Lepidium sativum, Amaranthus caudatus, A. cruentus, A. hypochondriacus, A. dubius, A. tricolor, A. lividus, виды рода Physalis L., Momordica charantia, Benincasa hispida, Cucumis metuliferus, Vigna unguiculata.

## Introduction

The domestication of many cultivated plants, including vegetable crops, often took place around the world in the form of introduction (Bazilevskaya, 1964). Some new species began to occupy leading positions in peasant farms, displacing the previous “favorites”, while others remained unclaimed. They are called “non-traditional crops” in the scientific agricultural literature.

The lack of planting material, insufficient awareness of the population (potential consumers) about nutritional and medicinal value of non-traditional crops and the lack of information about specific cultivation technology of these crops are considered to be possible reasons for insufficient use of non-traditional crops (Jena et al., 2018). In addition to these three reasons, another important factor in Russia is the presence of agrobiological traits and/or their expression, which limits the possibility of effective cultivation of such crops in the conditions of real agrocenosis in many regions of the country. A long growing season exceeding the duration of the frost-free period of many territories of the Russian Federation, poor resistance to low temperatures, high sensitivity of the generative sphere to environmental factors, susceptibility to diseases and pests, inadequacy of the quality of marketable products to consumer expectations also limit the potential of new crops and the possibility of their positioning as food products, including functional ones

## The problem of expanding production and the demand for new crops

The All-Union Institute of Plant Industry (now the All-Russian Institute of Plant Genetic Resources (VIR)), headed by N.I. Vavilov, played an outstanding role in the collecting and studying of the collection of vegetable plants which were new for Russia. Alot of new species were first included in the collection of the Institute with Vavilov’s active participation (Vavilov, 1987).

At present time the world collection of vegetable and melon crops of the Russian Federation, stored in the VIR, has more than 50 thousand samples belonging to 29 families, 145 genera, and 610 species. The status of the collection samples is as follows: 5.5 % are wild species and primitive forms; 34 % are landraces; 49 % – breeding (commercial) varieties; 11.5 % – different types of breeding lines and hybrids, including hybrid populations. The uniqueness of the collections of some vegetable crops reaches 80 %.Vegetable crops presented in the VIR collection belong mainly to 9 families:

- Brassicaceae Burn.;
- Solanaceae Juss.;
- Leguminosae Juss.;
- Cucurbitaceae Juss.;
- Alliaceae Borkh.;
- Apiaceae Lindl.;
- Amaranthaceae Juss.;
- Asteraceae Bercht. et J. Presl;
‒ Lamiaceae Martinov.

The diversity of these large taxonomic groups is exceptionally great in terms of the biochemical characteristics of the representatives of these families. Some of the species and crops that deserve priority inclusion in the introduction programs from the standpoint of their biochemical value and the possibility of use as functional foods (FF) are listed below. At the same time, the problem of expanding production of cold-resistant crops used as leafy vegetables (species from the Brassicaceae family and, partly, Amaranthaceae) in agricultural enterprises is largely associated with the lack of agricultural technologies and the availability (supply) of highquality seed material and, to a lesser extent, with their adaptive potential (e.g., preference of short-day and/or resistance to pathogens and pests), compared with traditional heat-loving vegetable crops. On the contrary, many introduced species of heat-loving vegetable plants from the Solanaceae, Cucurbitaceae, and Leguminosae families with narrow ecological plasticity are carriers of traits that prevent the scaling up their production in the regions of Russia (sensitivity to low temperatures and response to the day length, susceptibility to certain diseases, etc.) (Supplementary Material)^1^. Such species require significant breeding and genetic improvement for cultivation in a real agrocenosis

Supplementary Material is available in the online version of the paper: http://vavilov.elpub.ru/jour/manager/files/Suppl_Fotev_Engl.pdf


The Table provides basic information about the range of non-traditional vegetable crops presented in the State Register. The number of registered cultivars varies from 1 (naranjilla, kiwano) to 61 (Chinese cabbage), while the period of stay in it from the year of registration of the first cultivarup to 01.01.2021 varies from 12 (wax gourd, common chicory) to 78 years (leaf mustard). Most of the cultivars have been relatively recently bred and included in the State Register: the median of the year of inclusion falls on 2006–2020. The largest minus values of the coefficient of asymmetry by a year: –2.8, –2.4, –2, respectively, for mustard, Chinese cabbage, and bitter melon, illustrate a sharper increase in the number of inclusions in the State Register in recent years (which means an interest for them from consumers and breeders), compared with an earlier period – the year of registration of the first cultivar. On the contrary, the interest of breeders in the registration of amaranth cultivars has decreased in comparison with the previous period – the coefficient of skewness is 1.1. Leptokurtic (peaked) distribution by years of inclusion in the State Register cultivars of Chinese cabbage, mustard, and bitter melon with kurtosis indices, respectively, 10.3, 9.9, and 4.0 shows a significant increase in the number of registrations of breeding achievements for these species around the median indicator (year). The ratio of the total number of registered cultivars by crop to the number of years since the year of inclusion in the State Register of the first cultivar shows the relative degree of demand for the crop, although this may also indicate the lack of available intraspecific genetic diversity (biodiversity) necessary for its breeding improvement. The calculated “coefficient of demand for the crop” turned out to be the maximum for asparagus vigna (179), Chinese cabbage (107), and pakchoi (95).

**Table 1.**
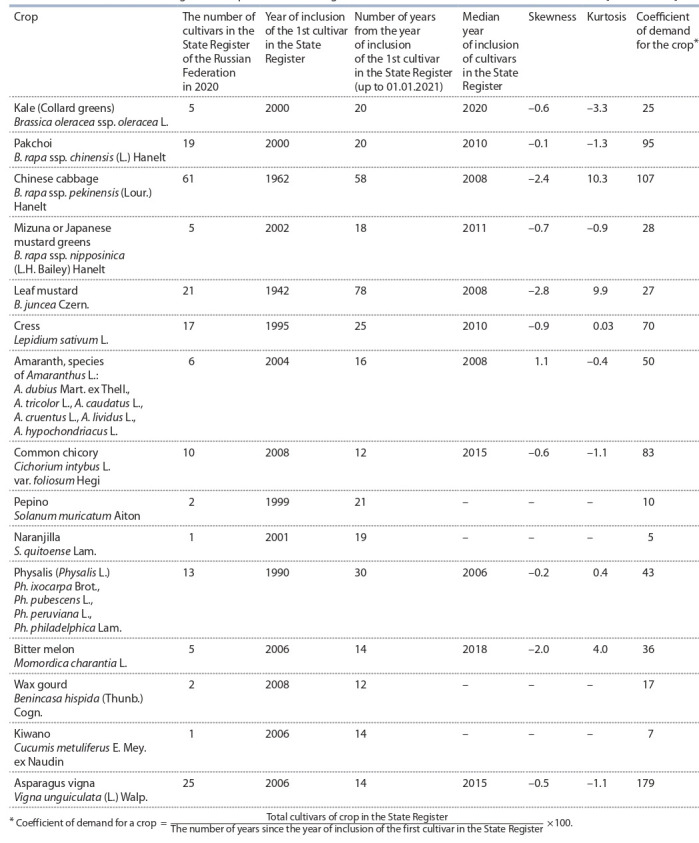
Assortment of non-traditional vegetable crops in the “State Register of Selection Achievements Authorized for Use” [National List]

Below, as an example of the choice of the object of introduction and selection, we will consider information about the three most promising, from our point of view, crops.

Asparagus vigna (Vigna unguiculata (L.) Walp.) is a valuable vegetable crop that could be used as a functional food (Fotev et al., 2019). Its cultivation in Russia is limited due to high heat demand, negative reaction of many cultivars for a long day, and susceptibility to some pathogens. The collection of cowpea VIR includes 4092 specimens of 9 species of the genus Vigna Savi (Vishnyakova et al., 2019). As of 01.01.2021, 25 Russian cultivars of V. unguiculata are included in the State Register.

**Fig. 1.**
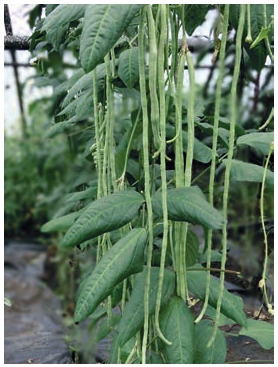
Asparagus vigna, cultivar Sibirskiy razmer. Photo by Yu.V. Fotev.

Cold hardiness is a complex trait in many crops. There are different methods of evaluating cold resistance. For example, a close positive correlation was found between the resistance to low temperatures of microgametophyte and sporophyte in tomato cultivars (Kilchevsky, Pugacheva, 2002). According to V.V. Vinogradova (1988) “when adapting tomato to low temperatures, the most effective assessment of cold resistance is the method of pollen germination in the solution of 15 % sucrose and H3BO3 (100 mg/l) at 6–10 °C” (p. 78). On a solution of a synthetic osmotically active substance – polyethylene glycol with a molecular weight of 6000 (PEG 6000), which does not participate in the metabolism of plant cells (Steuter et al., 1981), species, varieties, and interspecific hybrid forms of tomato combining resistance to low and high temperatures for stages of pollen germination in vitro were selected (Fotev, 2013). To assess the resistance of different samples of cowpea to low temperatures, it is advisable to evaluate the growth response of pollen in vitro also on a PEG 6000 solution at a concentration of 20 % with boric acid 0.006 % (Fotev, Belousova, 2013). In the Central Siberian Botanical Garden (hereinafter CSBG), the highest indices of cold resistance in the form of the ratio of pollen germination at low (10 °C – 24 h) temperature to the same index at 25 °C for 3 h were observed in V. unguiculata samples: Lulin (87 %), Zinder (65 %) and Sibirskiy razmer (46 %) (Fig. 1).

In addition, the cultivars of asparagus cowpea, Sibirskiy razmer (see Fig. 1), and Yunnanskaya, bred in Russia have a neutral reaction to day length.

Selected forms that showed a high level of resistance to Botrytis cinerea Pers. and Sclerotinia sclerotiorum (Lib.) de Barywere selected in the CSBG: forma 901, forma No. 323 [striped], Early Prolificacy Xiao Bao #2, F1 (Early Prolificacy Xiao Bao #2×Sibirskiy razmer) and F3 (Early Prolificacy Xiao Bao #2×Sibirskiy razmer) (Fotev, Kazakova, 2019).

Wax gourd (Benincasa hispida (Thunb.) Cogn.) originates from Indochina and is widely cultivated in India, Japan, China, and many other tropical countries. Wild wax gourds have small fruits (<10 cm in length), while most cultivars produce giant fruits (up to 80 cm in length and weighing over 20 kg).

Wax gourd fruits contain vitamins, flavonoids, triterpenoids, and metabolites that can be used in the treatment of various diseases. The plant is used as a tonic for the brain, heart disease, and nosebleeds (Biradar et al., 2016). This crop can be seen as avaluable FF.

**Fig. 2.**
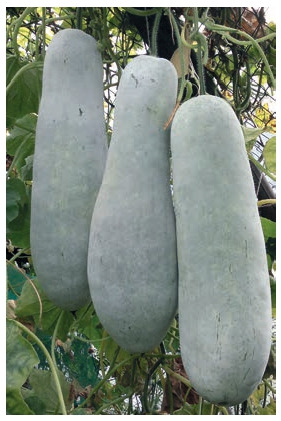
Wax gourd, cultivar Akulina. Photo by Yu.V. Fotev.

The first cultivar in Russia of wax gourd – Akulina (Fig. 2) was created in the CSBG. The direction for the improvement of the crop can be the breeding of more cold-resistant cultivars, gynoecious forms, and F1 hybrids based on them.

**Fig. 3.**
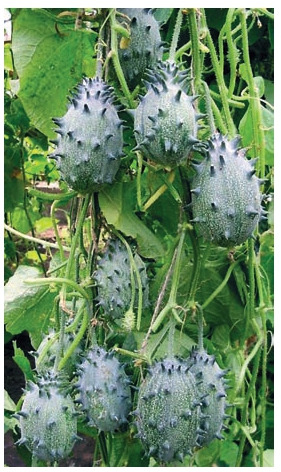
Kiwano, cultivar Zeleniy drakon. Photo by Yu.V. Fotev.

Kiwano (Cucumis metuliferus E. Mey. ex Naudin) is a vegetable crop, the fruits of which can be stored for up to six months under normal (“room”) conditions. Only one cultivar – Zeleniy drakon (Green Dragon) (Fig. 3) – included in the State Register is bred in the CSBG and characterized by a short period from germination to fruiting and high productivity in the outdoor conditions in the south of Western Siberia and greenhouses.

Kiwano alkaloids have a protective effect on both the liver and kidney tissue (Anyanwu et al., 2014) and antiviral properties against Newcastle disease caused by a virus from the family Paramyxoviridae, a dangerous pathogen for birds (Anyanwu et al., 2016).

Kiwano fruit tastes good but contains a lot of seeds. The use of parthenocarpy can solve this problem. It is known that a short day (Lim, 2012) and low temperatures (Benzioni, 1997) results in the formation of parthenocarpic fruits in this crop.

In addition, kiwano plants can serve as good rootstocks for watermelon against rootworm nematode from the genus Melodogyne Goeldi (Kyriacou et al., 2018) and for melon (Cucumis melo), due to resistance to nematodes and fusarium (Guan et al., 2014 ).

## Conclusion

As discussed above, the species and forms of vegetable crops that are promising for the Russian Federation were considered for introduction and subsequent breeding. Particular attention is paid to the species – potential sources of high functional food ingredients (FFI) content. These primarily include species of the Brassicaceae family: B. oleracea ssp. oleracea, B. oleraceae var. alboglabra, B. rapa ssp. chinensis, B. rapa ssp. narinosa, B. rapa ssp. nipposinica, B. rapa ssp. rapa, B. juncea, Cochlearia officinalis ssp. arctica, Lepidium sativum; Amaranthaceae: A. caudatus, A. cruentus, A. hypochondriacus, A. dubius, A. tricolor, A. lividus; Solanaceae: species Physalis L.; Cucurbitaceae: Momordica charantia, Benincasa hispida, Cucumis metuliferus; Leguminosae: Vigna unguiculata. The biological characteristics of introduced species limiting the scale of production of specific introduced crops in Russia are indicated. It is proposed the methodological approach for evaluation of resistance to low temperatures with the use of its assessment in the phase of a mature male gametophyte of Vigna unguiculata in vitro as an example.

The trait “high content of FFI” in a crop must be specified taking a 2–4 times excess of the content of individual FFI or their complex in the cultivar transferred to the State variety testing over the crop standard (reference) values as a basis. Such cultivars should be included in the State Register of Selection Achievements Authorized for Use in a separate list. The purpose of such allocation is to ensure the future interest of producers, investors, and business structures in the sale of FF vegetable products on the poor market of vegetables of the Russian Federation.

To increase the efficiency of introduction and breeding, it is proposed to use the index of the ratio of the total number of registered cultivars by a crop to the number of years since the year of inclusion to the State Register of the first cultivar as a characteristic of the degree of demand for the crop.

## Conflict of interest

The authors declare no conflict of interest.
